# The two dragons of cognition: recursive condensation for predictive processing

**DOI:** 10.3389/fncom.2026.1778902

**Published:** 2026-03-23

**Authors:** Xin Li

**Affiliations:** Department of Computer Science, University at Albany, Albany, NY, United States

**Keywords:** Memory-Amortized Inference (MAI), parity alternation principle, predictive processing, Savitch's theorem, topological condensation and expansion, Topological Trinity Transformation (TTT), Urysohn's Lemma, Spontaneous Slow Oscillations (SSO)

## Abstract

Computation separates time from space: nondeterministic problems are exponential in time (the “Time Dragon”) but polynomially simulable in space (the “Space Dragon”), as formalized by Savitch's theorem (NPSPACE⊆PSPACE). We propose that the brain physically instantiates this theorem through Recursive Condensation, a topological mechanism that converts intractable high-dimensional search into efficient low-dimensional navigation. Drawing on Urysohn's Lemma, we demonstrate that separability is a property of connectivity, not volume; a stable decision boundary exists independent of ambient dimension provided the underlying manifolds are topologically disjoint. To manufacture this disjointness, the cortex employs a parity alternation strategy: it alternates between odd-parity metric expansion (exploratory search) to untangle local geometry, and even-parity topological contraction (closure/condensation) to lock in validated invariants. This cycle acts as a biological “Savitch Machine,” mediating a Topological Trinity Transformation (TTT), *Search*→*Closure*→*Navigation*, that compiles high-entropy exploration paths into low-energy quotient tokens. Under Memory-Amortized Inference (MAI), the cortex slays the Space Dragon by collapsing vast state spaces into compact metric singularities, and tames the Time Dragon by memoizing these traversals as structural priors. Evolution's “cheat code,” linear cortical growth yielding exponential cognitive gain, emerges as a physical law of topological inference: exponential search in time becomes polynomial reuse in space via recursive metric collapse.

## 1 Introduction

A central puzzle in cognitive science and theoretical machine learning is how biological intelligence achieves *exponential* problem-solving abilities using only *polynomial* increases in physical substrate. Across mammals, neocortical surface area grows roughly linearly (Buzsaki, 2006), yet behavioral sophistication, planning depth, counterfactual reasoning, and social inference, grows superlinearly. How does a resource-limited biological system acquire the functional equivalent of exponential search? We propose that the answer lies in a dual confrontation with two fundamental constraints: the “Time Dragon” of combinatorial explosion and the “Space Dragon” of high-dimensional sparsity.

### 1.1 The two dragons: complexity and dimensionality

High-level cognition requires navigating a branching tree of counterfactual futures (*If I take action A, what becomes possible?*). Computationally, this faces the **Time Dragon**: nondeterministic search (NPSPACE), where the number of branches grows exponentially with depth (Minsky, 1967). Simultaneously, sensory processing faces the **Space Dragon**: the Curse of Dimensionality (Bellman, 1957). In the raw sensory manifold (e.g., the optic nerve with *D*≈10^6^), the volume of the space expands exponentially, rendering naive metric inference impossible due to data sparsity. Standard approaches treat these as separate problems, solving the former with heuristics and the latter with geometric kernels, but we argue the brain solves both via a single topological mechanism.

### 1.2 The bridge: from searching mazes to rolling in bowls

**Figure 1:**
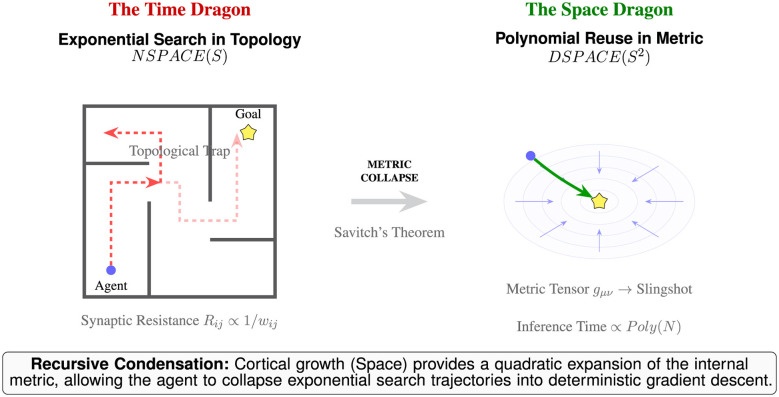
Recursive condensation as a complexity converter. This diagram illustrates the fundamental shift from topological search to metric navigation. The Time Dragon **(Left)**: represents the environment as a topological maze (NSPACE). In this regime, the agent is governed by graph-based connectivity where walls represent infinite synaptic resistance (*R*_*ij*_). Without a metric, the search for the goal is non-deterministic and exponential, leading to topological traps where local gradients vanish or point toward dead ends. The cheat code **(Center)**: inspired by Savitch's Theorem, the cortex utilizes linear growth in physical space (*S*) to build a quadratic representation of the internal metric (*S*^2^). This transformation, metric collapse, is the mechanism by which the brain solves the maze before entering it. The Space Dragon **(Right)**: represents the internalized metric bowl (DSPACE). Through GHL, the hippocampal-cortical circuit extracts the principal axes of the maze's fluidity. It warps the internal geometry (the Riemannian tensor *g*_μν_) to collapse the distance along the escape route. The result is a metric slingshot where complex path-finding is reduced to a deterministic, polynomial-time descent (inference) toward a global attractor.

This dual challenge is best understood through the **Maze-Bowl Analogy** (Figure 1). When the brain encounters a novel, high-dimensional problem, it is effectively a time dragon trapped in a topological maze; navigation is a non-deterministic search through a branching tree of corridors, where every dead end consumes metabolic time and energy. However, if the brain can utilize its cortical real estate (the “Space Dragon”) to represent the environment not as a list of edges, but as a warped Riemannian manifold, the problem undergoes a phase transition. As described by *Savitch's Theorem* (Savitch, 1970), the brain trades a quadratic expansion of representation space to collapse the search time. By learning the principal axes of fluidity within the maze, the cortex warps the internal metric until the complex maze becomes a simple, convex *bowl*. In this condensed geometry, the Search phase is bypassed: the agent no longer navigates a sequence of choices but simply follows a deterministic gradient, a Metric Slingshot, where the goal becomes a singular, reachable attractor.

### 1.3 The solution: recursive condensation

We propose that the cortex circumvents these barriers via *Recursive Condensation*: a strategy that transforms the global search problem into a hierarchy of local topological decisions. Drawing on Urysohn's Lemma (Urysohn, 1925), we demonstrate that effective separability is a property of *connectivity*, not volume. A stable decision boundary exists independent of ambient dimension *D*, provided the underlying representations are topologically disjoint (closed sets). The brain actively manufactures this disjointness through the **Parity Alternation Principle**: (1) *Odd Parity (Expansion):* the system performs a metric lift (e.g., pyramidal integration), exploring the open neighborhood of the input. This corresponds to the *Search* phase of inference. (2) *Even Parity (Contraction):* the system applies a metric collapse (e.g., lateral inhibition), condensing the neighborhood into a compact, zero-margin token. This corresponds to the *Closure* phase. By recursively applying this cycle, the brain compiles high-entropy exploration paths into low-energy navigation tokens, effectively “slaying the Space Dragon” by collapsing the ambient dimension at every step.

### 1.4 The biological Savitch machine

This topological serialization provides the physical hardware for *Savitch's Theorem* (NPSPACE⊆PSPACE) (Savitch, 1970), which proves that any problem solvable by nondeterministic parallel search can be simulated by a deterministic machine with polynomial memory, provided it can recompute (or recall) intermediate steps. We define this process as the *Topological Trinity Transformation* (TTT): $\text{Search (Time)}\overset{\text{Closure}}{\to }\text{Navigation (Space)}$. Under *Memory-Amortized Inference* (MAI), the cortex acts as a memoized Savitch Machine. Instead of discarding the intermediate steps of the recursion, it consolidates them into the synaptic weights of cortical columns (Mountcastle, 1957). This allows the system to tame the *time dragon*: it converts the exponential cost of searching a path into the polynomial cost of retrieving a pre-computed token.

### 1.5 Evolution's scaling advantage

This relationship, linear hardware ⇒ exponential cognitive leverage, creates a powerful evolutionary feedback loop. Each incremental increase in cortical area adds more Urysohn operators (columns), increasing the number of reusable quotient tokens available for composition. The neocortex thus becomes an evolutionary cheat code: by enforcing topological closure on inference dynamics, it allows a finite biological substrate to emulate the connectivity of an infinite state space. This paper develops the theoretical and computational foundations of this principle. Our contributions are:

1. **Formalization of recursive condensation:** we introduce *Recursive Condensation* as a topological mechanism that solves the Space Dragon (Curse of Dimensionality) by applying the *Parity Alternation Principle*, alternating between odd-parity metric expansion and even-parity topological contraction, to manufacture the disjoint closed sets required by Urysohn's Lemma.
2. **The biological Savitch machine:** we establish that the brain acts as a physical instantiation of Savitch's Theorem (NPSPACE⊆PSPACE). We show that by memoizing intermediate search steps into structural tokens, the cortex implements a *Memory-Amortized Inference* (MAI) engine that tames the Time Dragon, converting exponential parallel search into tractable serial retrieval.
3. **The Topological Trinity Transformation (TTT):** we derive the TTT (*Search*→*Closure*→*Navigation*) as the dynamical execution of the constructive Urysohn proof. We prove that this sequence satisfies the *Principle of Least Computational Action* (PLCA), compiling high-entropy exploration paths into low-energy geodesic shortcuts.
4. **Neurobiological instantiation:** we interpret the neocortical architecture through this topological lens, identifying the *Polychronous Neural Group* (PNG) as the temporal search operator (Odd Parity/β_1_) and the *Cortical Column* as the spatial navigation operator (Even Parity/β_0_). We map the transition between them to Spike-Timing-Dependent Plasticity (STDP) acting as a metric collapse function.
5. **Unified scaling law:** we demonstrate how these mechanisms explain the evolutionary cheat code of mammalian intelligence: we derive a scaling law showing that linear growth in cortical surface area yields exponential gains in representational capacity by recursively exploiting the metric independence of topological invariants.

## 2 The two dragons: time vs. space

**The setting**: at the dawn of cognitive science, researchers sought a mechanistic understanding of how intelligence could possibly work. Minsky articulated the central challenge as the *search problem* (Minsky, 1967): any intelligent act (e.g., planning a chess move or constructing a sentence) appears to involve a vast combinatorial expansion of “what-if” possibilities (e.g., counterfactual reasoning Bottou et al., 2013). Each potential decision spawns multiple counterfactual futures, forming an exponential tree of depth and breadth so immense that even modest tasks imply astronomical complexity (~10^120^ possible sequences in chess). This was the original dragon of cognition, the realization that *thought itself is an exponential search problem*.

**Dragon I: time (P vs. NP)**: the time question asks: *Can we find a solution as fast as we can verify one?* Let P denote problems solvable in polynomial time by a deterministic Turing machine, and NP those whose solutions can be verified in polynomial time. The central open problem asks whether P = NP. Consensus in theoretical computer science strongly favors P≠NP, i.e., exhaustive “what-if” search generally cannot be replaced by equally fast deterministic solution methods (Cook, 1971; Karp, 1972; Sipser, 2012). This *time* barrier is believed to be fundamental.

**Dragon II: space (NPSPACE vs. DSPACE)**: the space question asks: *Does a nondeterministic “what-if” search inherently require exponentially more memory than a step-by-step deterministic simulation?* Let NSPACE(*s*(*n*)) (resp. DSPACE(*s*(*n*))) be the class of languages decidable using *O*(*s*(*n*)) space by a nondeterministic (resp. deterministic) machine. Savitch's Theorem shows that nondeterministic space can be *serialized* with only quadratic overhead: NSPACE(*s*(*n*))⊆DSPACE*s*(*n*)^2^ for*s*(*n*)≥log*n*. As a corollary, NPSPACE = PSPACE = DSPACE(*n*^*O*(1)^), so the space dragon was already slain in 1970: *nondeterministic* polynomial space offers no more power than *deterministic* polynomial space (Savitch, 1970; Sipser, 2012).

### 2.1 Two failed factions: cognitivism vs. embodiment

As cognitive science matured through the late twentieth century, two great schools of thought emerged, each confronting Minsky's dragon of exponential search, and each failing in the opposite direction. Both camps sought to explain how an intelligent system could act coherently in a world too complex to model exhaustively. Their divergence defined decades of AI research before the deep learning revolution.

#### 2.1.1 The mapmakers (GOFAI/cognitivism)

The first faction viewed intelligence as *representation*. Inspired by logic and symbolic reasoning, the Good Old-Fashioned AI (GOFAI) paradigm sought to construct a perfect internal *map* of the external world (Minsky, 1961). Cognition was modeled as rule-based inference over an explicit, propositional knowledge base. In principle, if the map were accurate enough, intelligent behavior would follow as theorem-proving or search within that representation. The mapmakers built agents that were intricate but inert. Their systems were too brittle and fragile to survive in the open world. Every real-world change demanded a costly recomputation of their internal model before taking any action. Trapped in their own symbolic universes, these agents became paralyzed by the very complexity they sought to tame, overwhelmed by Minsky's combinatorial dragon before taking their first step.

#### 2.1.2 The rebels (Brooks/embodiment)

The second faction rose in rebellion. Led by (Brooks 1991), the embodied or reaction-based movement rejected internal models altogether, proclaiming “*Intelligence without representation*.” They argued that the world itself serves as the best model of the world; perception and action should be tightly coupled, producing emergent intelligence through direct environmental engagement. The Rebels' robots were fast, robust, and adaptive in the “here and now,” but their success came at the cost of foresight. They could react, but not imagine. Without an internal model, they lacked the capacity for counterfactual reasoning, long-term planning, or reflection. Unable to ask “what if?”, they were condemned to a perpetual present, immune to Minsky's dragon, yet forever grounded to the surface of the immediate world.

#### 2.1.3 The dragon slayer with a statistical oracle

By the turn of the century, the field stood at an impasse. The mapmakers had minds without bodies; the Rebels had bodies without minds. To act intelligently, an agent must possess an internal model, but possessing such a model guarantees exposure to exponential combinatorics. Intelligence appeared caught between two impossibilities: *the paralysis of perfect representation and the mindlessness of pure reaction*. The early 2010s marked a decisive turning point in AI with the resurgence of *deep learning*, neural networks trained end-to-end on large datasets using gradient-based optimization. This approach culminated in the rise of *Large Language Models (LLMs)* (Brown et al., 2020), massive transformer architectures trained on trillions of tokens. LLMs demonstrated that scale itself functions as a new organizing principle (LeCun et al., 2015): **depth as abstraction**, **data as world model**, and **self-supervision as bootstrapping**. The LLM paradigm is the ultimate expression of Minsky's original heuristic solution. It is a brilliant guesser or a *statistical oracle*. An LLM solves the exponential search problem by avoiding it. Through massively parallel pattern recognition on vast data, it learns the statistical correlations of its domain so well that it can, in a single feed-forward pass, produce the most probable answer (Vaswani et al., 2017). It is a black-box that intuits what comes next, without a formal, step-by-step simulation of why. It is the world as its own best model, where the world is the training data (but no understanding).

### 2.2 The three clues: topology, complexity, and neuroscience

**Figure 2:**
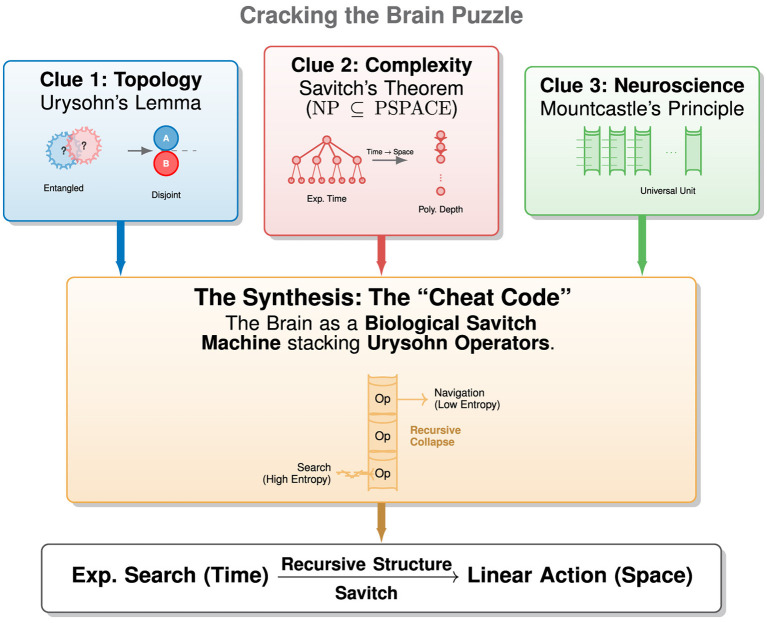
The convergence of the three clues: solving the paradox of intelligence. This diagram illustrates how three disparate discoveries unify to explain the scaling power of the neocortex. **(Left)** (Topology): Urysohn's Lemma demonstrates that the Curse of Dimensionality is solvable via topological closure; separability is a property of connectivity, not ambient volume. **(Center)** (Complexity): Savitch's Theorem proves that exponential search paths (Time) can be simulated by recursive structural depth (Space). **(Right)** (Neuroscience): Mountcastle's Principle identifies the cortical column as a universal, recursive hardware unit. Synthesis: we propose that the brain combines these principles to function as a *Biological Savitch Machine*. By stacking cortical columns acting as *Urysohn Operators*, the cortex implements the evolutionary cheat code: it linearizes the exponential complexity of environmental search by recursively condensing it into a polynomial spatial structure.

Decades pass, and Minsky's dragon, the exponential explosion of what-if possibilities, remains undefeated. AI oscillates between symbolic paralysis and reactive myopia, while the deeper question of *how intelligence circumvents exponential search* stays unanswered. To resolve this paradox requires looking beyond any single discipline. We propose that the answer lies in the convergence of three distinct discoveries, each holding a fragment of the same puzzle (Figure 2).

**Clue 1: the topological guarantee (Urysohn's Lemma)**: in the abstract realm of topology, Pavel Urysohn proved a fundamental result about separability: distinct concepts can be separated by a continuous function if and only if they are *closed* and *disjoint* sets (Urysohn, 1925). Crucially, this lemma is dimension-agnostic. It implies that the Curse of Dimensionality is not an intrinsic barrier to intelligence, but a geometric one (or the curse of Lipschitz Continuity Rudin, 1976). If a system can actively deform its representations, collapsing fuzzy, entangled manifolds into compact, disjoint sets, it can maintain linear separability regardless of the ambient complexity. Urysohn provides the *mathematical license*: the brain does not need to fill the void of high-dimensional space; it only needs to manufacture the topological conditions (disjoint closure) that make the void irrelevant.

**Clue 2: the computational shortcut (Savitch's theorem)**: in complexity theory, Walter Savitch proved that the Time Dragon (exponential branching) is not invincible. His theorem, NPSPACE⊆PSPACE, demonstrates that any problem solvable by nondeterministic parallel search can be simulated by a deterministic machine using only polynomial space, provided it exploits recursion (Savitch, 1970). The trade-off is specific: one can trade *exponential time* for *structural depth*. By identifying and storing intermediate midpoints (recurrence), a serial system can emulate a massively parallel one. Savitch provides the *algorithmic blueprint*: the brain can tame the combinatorial explosion not by growing faster, but by growing *deeper*, converting temporal search paths into spatial memory structures (Hasson et al., 2015).

**Clue 3: the physical engine (Mountcastle's principle)**: in neuroscience, Vernon Mountcastle peered into the neocortex and found, to his astonishment, not a multitude of specialized circuits but the *same circuit repeated everywhere*. Each cortical column, whether in vision, hearing, or planning, shares the same canonical six-layered architecture (Mountcastle, 1957). Mountcastle identified the *physical engine*: a universal, modular operator. For decades, this uniformity of cortical columns was a mystery: a key without a lock or a structure without a function (Horton and Adams, 2005). Why would the brain use the exact same circuit to process sensory stimuli and motor decisions?

**The synthesis: the cheat code**: we propose that these three clues describe the same phenomenon at different levels of abstraction. The brain is a **Biological Savitch Machine** (Clue 2) built from a recursive stack of **Cortical Columns** (Clue 3). The function of this universal column is to act as a **Urysohn Operator** (Clue 1): it takes an entangled, high-entropy input (Search), applies a metric collapse to enforce topological closure, and outputs a separated, low-entropy token (Navigation). Evolution's cheat code was not to solve but to exploit the equivalence by exponential growth of the neocortex (Buzsaki, 2006). By stacking these Urysohn operators, the cortex linearizes the exponential complexity of the world (Simon, 1962): *Exponential Search (Time)*→*Recursive Structure (Space)*→*Linear Readout (Action)*.

## 3 Recursive condensation and topological trinity transformation

Having defined the twin constraints of the Time Dragon (combinatorial explosion) and the Space Dragon (curse of dimensionality), we now derive the mathematical and dynamical principles by which the brain resolves them. We term this solution *recursive condensation*: a strategy that recursively collapses high-dimensional, entangled manifolds into low-dimensional, topologically separated quotient spaces.

### 3.1 The topological solution: Urysohn's Lemma

The fundamental barrier to inference in high-dimensional spaces is the sparsity of data, often formulated as the Curse of Dimensionality (CoD) (Bellman, 1957). In a raw sensory manifold $X\subset {\mathbb{R}}^{D}$ (where *D*≫10^5^), the Euclidean distance between any two random points approaches a constant, rendering standard metric-based classification unstable. However, topology offers an escape route: **Urysohn's Lemma** (Urysohn, 1925) offers the guarantee for continuous separability.

** Theorem 1 (Urysohn Separability)**. Let A,B⊂X be disjoint closed sets in a normal space. Then there exists a continuous separating function *f* such that the decision boundary lies within the open margin U=X\(A∪B).

Theorem 1's guarantee is *dimension-agnostic*. It depends entirely on the *connectivity* or *connectedness*, not volume. As long as the neural representation of a concept (Set *A*) can be topologically hardened into a closed set that does not intersect with the representation of a competing concept (Set *B*), a linear readout mechanism exists regardless of the ambient dimension *D*. Based on this rigorous result, the computational challenge of the brain, therefore, is not to fill the void of high-dimensional space, but to actively manufacture the topological condition of *disjoint closure*.

** Remark 1 (The topological paradox of dimensionality)**. A counterintuitive corollary to Urysohn's Lemma arises regarding the role of high-dimensional spaces. While typically feared as the Curse of Dimensionality in statistical learning (Geman et al., 1992), topologically, high dimensions represent a Blessing of Connectivity. We offer two lines of reasoning about the dimensionality paradox next. **1. Geometry vs. topology**. Consider the constraints of separation in lower dimensions: (a) **1D (the line):** if set *A* is at *x* = 0 and set *B* is at *x* = 10, an observer at *x* = 5 acts as a wall. The separating set is a point that blocks the path; connectivity is easily broken. (b) **High-D (the cloud):** in ℝ^*n*^ where *n*≫1, the degrees of freedom to separate two disjoint manifolds increase effortlessly. There are effectively infinite continuous deformations available to slide a separating sheet between two sets without intersection. An intriguing paradox emerges: *Geometry* views high dimensions as sparse and intractable (volume explosion), whereas *Topology* views them as spacious and flexible (connectivity freedom). **2. The Kernel trick vs. metric collapse**. If high dimensions facilitate separation, why is the CoD a problem? It depends on the strategy used to exploit those dimensions. (a) **Strategy A: The Kernel Trick (Space Inflation)**. Kernel methods such as support vector machines (SVMs) (Vapnik, 1998) successfully overcome the CoD by implicitly mapping inputs to a higher-dimensional feature space (ϕ:ℝ^*d*^ → ℝ^∞^). By inflating the space, they render nonlinear manifolds linearly separable. *The Limitation:* While topologically valid, this approach focuses on defining the **boundary** (the shoreline between manifolds). It requires maintaining a set of support vectors to define this complex surface. Kernel trick solves separability by *increasing* representational complexity. (b) **Strategy B: metric collapse (space condensation)**. The framework proposed here takes the inverse approach. Instead of inflating the space to find a hyperplane, it employs the Urysohn function to **collapse** the manifold. Rather than balancing a plane between two complex surfaces, one can effectively implement the distance ratio: $f\left(x\right)\approx \frac{d\left(x,C_{A}\right)}{d\left(x,C_{A}\right)+d\left(x,C_{B}\right)}$. By collapsing the manifolds to their centroids or prototypes ($C_{A},C_{B}$), we force the problem to depend only on the 1D distance to the prototype. **Conclusion:** Kernel methods exploit the blessing by expanding *x* until walls vanish. Metric collapse exploits the blessing by contracting *A* and *B* until the empty space is irrelevant. The former is a *boundary-oriented* solution; the latter is a *core-oriented* solution, completely bypassing the need to tile the ambient dimensions.

#### 3.1.1 The Turing-Urysohn duality: from mechanism to topology

While Turing's (1952) work on Reaction-Diffusion and Urysohn's (1925) work on Topology originate from distinct domains, they represent complementary facets of the same morphogenetic imperative: the *creation of boundaries*. We posit that Turing provides the *physical mechanism* necessary to satisfy Urysohn's *topological preconditions*. In a homogeneous medium (sensory soup), Turing's activator-inhibitor dynamics induce spontaneous symmetry breaking, effectively manufacturing two disjoint closed sets (*A* and *B*) by amplifying local signals (Closure) and suppressing neighbors (Disjointness). This physical segmentation creates the required geometric substrate for Urysohn's Lemma, which then *licenses* the existence of a continuous function, manifested physically as a chemical concentration profile or neurally as a semantic gradient, to cleanly separate these regions. In the context of biological intelligence, this duality explains the transition from continuous entropy to discrete thought: neural lateral inhibition (the Turing engine) performs the “Urysohn Cut,” collapsing the fuzzy sensory manifold into distinct, computable concepts.

### 3.2 The parity alternation principle: algorithmic topology

How does a biological system ensure that its representations are *closed* and *disjoint* in a noisy, continuous world? The real world presents a continuum, while the separability Urysohn's Lemma promises, demands discrete topological separation. We propose that the cortex bridges this gap through the **Parity Alternation Principle**. Inference is not a static state but a rhythmic oscillation between two topological phases (e.g., sleep-vs-wake Hinton et al., 1995), governed by the duality of homology groups. To understand why this oscillation is necessary, we must distinguish the *static guarantee* from the *dynamic mechanism*.

#### 3.2.1 The guarantee vs. the engine

Urysohn's Lemma provides the license for intelligence, but it does not manufacture the fuel. (1) **Urysohn (the static guarantee):** if two concept supports A,B⊂Z are disjoint closed sets in a normal space, there exists a continuous gate f:Z→[0,1] such that *f*|_*A*_ = 0 and *f*|_*B*_ = 1. This guarantees that a stable soft separator *exists*. (2) **Parity alternation (the dynamic mechanism):** existence is useless without construction. Parity alternation is the engine that actively manufactures the topological conditions required by Urysohn. By oscillating between two phases (odd/expansion vs. even/condensation), the system actively creates closed supports (via consolidation) and pushes them apart (via conflict detection). It forces the condition *A*∩*B* = ∅ to become true and stay true.

#### 3.2.2 The deeper fit: the cortex simulates Urysohn's proof

The most profound insight is that the standard mathematical proof of Urysohn's Lemma is, itself, a parity alternation algorithm. The proof constructs a separating function by building a family of nested open sets {_*U*_*r*_}*r*∈ℚ_ such that: $A\subset U_{0},\;\bar{U_{r}}\subset U_{s}\text{for}r<s,\;\bar{U_{1}}\cap B=\emptyset$. The proof proceeds by alternating two distinct operations: (1) *Open neighborhood selection (expansion):* find an open set *U* that covers the concept *A*. This effectively proposes a region of influence. (2) *Closure enforcement (condensation):* take the closure $\bar{U}$ and ensure it sits strictly inside the next layer, disjoint from *B*, which validates the region and creates a solid boundary. We propose that the *Parity Alternation Principle* is the algorithmic instantiation of this topological construction. The brain does not merely satisfy the theorem; it *executes the proof steps* in time.

#### 3.2.3 Machine learning interpretation: the phasic cycle

In the language of latent space dynamics, this manifests as a rhythmic update rule for maintaining the disjointness of memory supports (*A*_*t*_). (a) *Odd phase (plastic/expansion):* this mirrors the Open-Set step. The system becomes plastic, exploring new trajectories and generating candidate regions *U* where new data wants to live. It detects topological conflicts (overlaps, parity inconsistencies) between the new input and existing memory structures. (b) *Even phase (rigid/condensation):* this mirrors the Closure step. The system becomes rigid, contracting validated trajectories into closed tokens (attractor basins or prototypes). This enforces the disjointness constraint: $\text{dist}\left({\bar{A}}_{\text{old}},{\bar{A}}_{\text{new}}\right)>\epsilon$. Overlaps are resolved by pushing conflicting data into boundary adjustments or splitting components. Once this cycle stabilizes, the supports *A* and *B* are mathematically prepared for the Urysohn License, which enables: (1) *Continual learning* (Wang et al., 2024): updates are routed to *B* via the continuous gate without rewriting the closed support of *A* (solving catastrophic forgetting). (2) *Out-of-distribution (OOD) detection* (Yang et al., 2024): novelty is strictly defined as distance-to-closed-support; if a point falls into the void between disjoint sets, it is rejected. (3) *Compute scaling:* the system replaces global discrimination with local indexing (gate-then-refine).

** Remark 2 (The Euler Thermostat)**. The interaction between these phases effectively regulates the “topological temperature” of the manifold via the Euler characteristic (Hatcher, 2005): $\chi =\underset{k}{\sum }{\left(-1\right)}^{k}\beta_{k}=\beta_{0}-\beta_{1}+\ldots \;$. By alternating between injecting entropy (Odd/β_1_) and extracting structure (Even/β_0_), the brain prevents both *overfitting* (too many disjoint points, high β_0_) and *entanglement* (too many unresolvable loops, high β_1_). To physically instantiate this topological regulation within the constraints of neural circuits, the abstract requirement of parity alternation must be mapped onto a concrete temporal processing cycle.

### 3.3 The topological trinity transformation (TTT)

**Figure 3:**
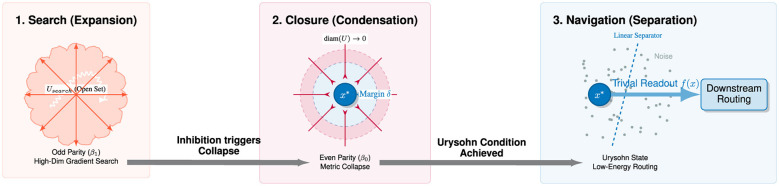
The Topological Trinity Transformation (TTT). This diagram illustrates the conversion of high-entropy sensory data into low-entropy invariant tokens. (1) Search (expansion): triggered by surprisal, the system enters a high-temperature Odd Parity state (β_1_), expanding a fuzzy open set *U*_*search*_ along gradient manifolds to interpret the input. (2) Closure (condensation): inhibitory feedback triggers a Metric Collapse (Even Parity, β_0_), driving the diameter of the candidate set to zero (diam(*U*) → 0). This creates a distinct topological token x^*^ surrounded by a safety margin δ, satisfying the disjointness condition. (3) Navigation (separation): once collapsed, the complex high-dimensional manifold is reduced to a point mass. The Urysohn separating function *f*(*x*) becomes a trivial linear readout structure, allowing for efficient low-energy routing to downstream modules.

The Parity Alternation Principle is operationally realized through a three-stage dynamical cycle we term the *Topological Trinity Transformation* (TTT). This cycle describes the conversion of raw sensory data into predictive invariants (Figure 3).

1. **Search (expansion):** the system enters the Odd Parity regime. Driven by prediction error (surprisal), neural activity propagates along high-dimensional gradients. This defines a candidate open set *U*_*search*_ representing the potential interpretation of the input.
2. **Closure (condensation):** this is the critical step often overlooked in purely associative models. To satisfy Urysohn's condition, the system must actively enforce disjointness. Inhibitory mechanisms (Even Parity) trigger a **Metric Collapse**, driving internal distances within the candidate manifold to zero: $\text{diam}(U)\overset{\text{Collapse}}{\to }0\Rightarrow U\to \{x^{*}\}.$. This physical deformation manufactures the necessary margin δ between the concept and the background noise, transforming a fuzzy probability cloud into a distinct topological token.
3. **Navigation (separation):** once the representation is condensed into a closed singularity, the system enters the Navigation regime. The Urysohn separating function *f*(*x*) becomes computationally trivial to evaluate (e.g., a linear readout), allowing for low-energy routing of the token to downstream areas.

#### 3.3.1 The Urysohn-TTT isomorphism

The TTT is not merely a heuristic; it is the dynamical execution of the constructive proof of Urysohn's Lemma. We can map the mathematical steps of the proof directly to the cognitive phases of the TTT (Table 1).

**Table 1:** The isomorphism between Urysohn's Lemma and the topological trinity transformation.

**Mathematical construction (Urysohn)**	**Cognitive mechanism (TTT)**
**Step 1: open set identification** Identify an open neighborhood *U* such that A⊂U⊂X\B. This defines the region of influence.	**Phase 1: search (expansion)** The brain enters the *Odd Parity* regime. Driven by surprisal, neural activity expands along high-dimensional gradients to identify the basin of attraction for the stimulus.
**Step 2: closure and normality** Construct the closure $\bar{U}$ and verify disjointness ($\bar{U}\cap B=\emptyset$). This ensures a valid topological boundary exists.	**Phase 2: closure (condensation)** The brain enters the *Even Parity* regime. Inhibitory feedback triggers Metric Collapse, suppressing competing representations to manufacture a safety margin δ>0.
**Step 3: functional/structural definition** Recursively interpolate nested sets to define the continuous function *f*(*x*).	**Phase 3: navigation (separation)** With the representation condensed to a token (x^*^), the complex manifold is treated as a point mass. The separating function becomes a trivial linear readout.

** Definition 1 (The Urysohn-TTT Isomorphism)**. We identify the stages of the Topological Trinity Transformation as the algorithmic implementation of Urysohn's construction.

#### 3.3.2 The payoff: navigation and the principle of least computational action

In our framework, navigation is effectively the biological evaluation of the Urysohn separating function. However, this is not merely a mathematical convenience; it is an energetic necessity. By manufacturing the topological conditions required by the lemma (closed disjoint sets), the brain ensures that the following *Principle of Least Computational Action (PLCA)* holds: *A cognitive system minimizes the metabolic and temporal cost of frequent inference by maximizing the topological separability of its latent representations*. It essentially trades *structural entropy* (during learning) for *inference efficiency* (during behavior). The above principle serves as the functional bridge between our three clues: (1) *The economic trade-off (linking to Savitch):* recall Clue 2 (Complexity), where Savitch's Theorem allows trading time for space. The TTT cycle implements this trade-off physically. (a) *High Action (Search/Closure):* the system expends significant energy to collapse the manifold and define the boundary. This corresponds to the recursive depth of the Savitch machine (Savitch, 1970). b) *Least action (navigation):* once the token is defined (*U* → {x^*^}), the system no longer needs to solve a high-dimensional optimization problem to classify the input. It simply checks the token's coordinate. The complex search problem is reduced to a simple lookup (Bellman, 1957). (2) *The quotient manifold (linking to topology):* mathematically, the Metric Collapse creates a quotient space X/~ (Hatcher, 2005), where all points inside the fuzzy cloud *U* are identified as equivalent. In the raw sensory manifold, moving from Concept A to Concept B requires navigating a complex, noisy, high-dimensional terrain. In the quotient manifold (the space of collapsed tokens), the path is a straight line (a geodesic). (3) *The biological reality:* standard neural networks often require constant high action, processing the full high-dimensional vector for every query. The brain, limited by ATP, cannot afford this (Bennett, 2023). By ensuring representations are disjoint (Urysohn), the brain enables downstream circuits (e.g., the Basal Ganglia) to execute decisions via simple linear summation (action selection) rather than complex non-linear integration. In summary, navigation is the state where the brain reaps the rewards of its topological labor. The system has successfully converted a *search problem* (exponential cost) into a *routing problem* (linear cost).

## 4 The predictive architecture: cortical columns as Urysohn operators

How does the biological substrate implement these abstract topological operations? We propose that the neocortex is not merely a feature extractor, but a hierarchical machine designed to perform *recursive condensation*. We reinterpret Mountcastle's columnar organization principle (Mountcastle, 1957) through the lens of our TTT, positing that the cortical column functions as a discrete *Urysohn operator*.

### 4.1 The column as a condensation chamber

Standard models of predictive processing view the cortical column as a statistical error-minimization unit (Bastos et al., 2012). We extend this view by defining the column topologically as a physical *quotient map* q:X→X/~, responsible for collapsing a high-dimensional, entangled sensory manifold into a low-dimensional, disjoint token.

#### 4.1.1 Bottom-up: the open set (search)

The input layer (Layer IV) receives high-dimensional thalamic or cortical drive. Topologically, this represents the *metric expansion* phase (odd parity). The input defines an open set *U*_*input*_: a fuzzy, probabilistic cloud of potential interpretations. In the language of PP, this is the *prediction error* signal, representing the divergence between the current sensory flow and the stored invariants.

#### 4.1.2 Top-down: the closed set (prior)

Feedback connections from higher cortical areas (targeting Layers I/VI) project the *closed set* ${\bar{U}}_{prior}$. This is the topological invariant or expectation. It acts as a constraint, defining the valid manifold M upon which the input should lie.

#### 4.1.3 The Urysohn cut: lateral inhibition

The computation happens in the supragranular layers (II/III). Here, lateral inhibition acts as the *Urysohn Cut*. By suppressing competing columns, the circuit actively enforces the condition $\bar{U}\cap B=\emptyset$.

- If the open input *U*_*input*_ successfully collapses into the closed prior ${\bar{U}}_{prior}$ (i.e., $U_{input}\subset {\bar{U}}_{prior}$), the column enters *navigation* mode. It emits a condensed token (Layer V/VI output) with zero error.
- If the collapse fails (leakage, $U_{input}⊄{\bar{U}}_{prior}$), the system generates a *topological prediction error*. This is not just a scalar magnitude difference, but a geometry failure, triggering a new *search* phase (Odd Parity) to find a better topological fit.

#### 4.1.4 Reinterpreting the canonical microcircuit via TTT

**Figure 4:**
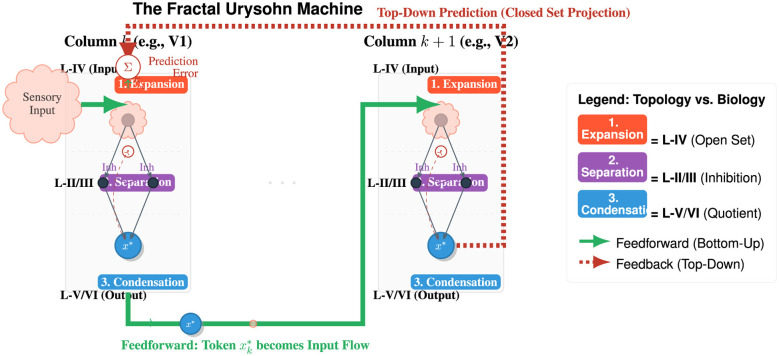
The cortex as fractal Urysohn machine. This diagram illustrates the mapping between the canonical cortical column and the topological condensation phases. 1) The vertical circuit (intra-columnar): processing moves from input expansion to output condensation. *Layer IV (expansion):* the column receives high-dimensional entangled input (Open Set *U*). *Layer II/III (separation):* lateral inhibition suppresses competing hypotheses, enforcing the disjointness condition ($\bar{U}\cap B=\emptyset$). *Layer V/VI (condensation):* the validated manifold is collapsed into a stable, low-dimensional token x^*^ (the Quotient Map). 2) The horizontal hierarchy (inter-columnar): the system is recursive. The condensed output token of Column *k* becomes the expansive input flow for Column *k*+1, effectively computing a sequence of quotient spaces $M_{0}\to M_{1}\to \ldots \;$. 3) Predictive flow: top-down feedback (Red) projects the Closed-Set expectations. Prediction error represents a topological mismatch: if the bottom-up flow (Green) falls outside the predicted closed support, the system reverts to high-entropy search.

Reframing the cortical column through the lens of the Topological Trinity Transformation fundamentally alters the interpretation of “precision weighting” in predictive processing. While standard models view the interplay between superficial and deep layers merely as error propagation modulated by synaptic gain, TTT posits that the microcircuit functions as a physical engine for *metric contraction*. In this view, superficial layers (L2/3) maintain the high-entropy *Search* phase, where “prediction error” effectively measures the homological void between sensory input and the prior manifold; conversely, deep layers (L5/6) encode the low-entropy *Structure*. The critical computation occurring between them is not simple subtraction but a thermodynamic phase transition (Figure 4), the lateral inhibition (Turing mechanism) in L2/3 performs the critical *Closure* (the Urysohn cut), physically collapsing the dimensionality of the search space. An update in the predictive model represents not just a shift in parameters, but a *topological surgery* that modifies the Betti numbers of the internal representation to resolve geometric ambiguity.

### 4.2 Recursive Savitch: hierarchical prediction

Standard PP formulations often face a computational bottleneck: predicting the exact pixel-level future at long time scales requires simulating a chaotic dynamical system, which encounters the *Time Dragon* (Lyapunov instability). We propose that the cortical hierarchy implements *memoized Savitch* to solve this. Instead of predicting the continuous trajectory *x*(*t*) for all *t*, the hierarchy predicts a sequence of *topological midpoints*.

1. **Higher columns (context):** predict the coarse-grained manifold $M_{context}$ (e.g., “Object Motion”). This sets the topological envelope for the layer below.
2. **Lower columns (detail):** do not need to search the entire state space. They only search for the geodesic path *within* the projected envelope $M_{context}$.

This recursively breaks the prediction horizon into manageable spatial chunks. The hierarchy $M_{0}\to M_{1}\to M_{2}$ corresponds to the recursion stack of Savitch's algorithm. By condensing temporal sequences into spatial tokens at each level, the brain converts the exponential difficulty of long-term prediction into a polynomial structural depth.

**Parity in plasticity: from flow to prior**. Finally, how does a transient search become a permanent prior? We argue that this phase transition is governed by the *parity alternation* of neural assemblies.

**Odd parity (β_1_): Polychronous Neural Groups (PNGs)**. The biological signature of the search phase is the **Polychronous Neural Group** (Izhikevich, 2006). PNGs are time-locked, spatiotemporal firing patterns. Topologically, a PNG is a **1-cycle** (a loop in time). It represents active inference, surprise, or the uncondensed flow of information. It is metabolically expensive and transient, corresponding to the NPSPACE search trajectory.

**Even parity (β_0_): columnar assemblies**. The biological signature of the prior is the stable **Columnar Assembly**. Topologically, this is a **0-cycle** (a point in space). The transition is mediated by *Spike-Timing-Dependent Plasticity* (STDP) (Caporale and Dan, 2008). When a PNG loop repeats reliably (validation), STDP reinforces the synaptic backbone, effectively collapsing the temporal delay loop into a simultaneous spatial ensemble.


$$\underset{\beta_{1},\text{High Entropy}}{\underset{︸}{\text{PNG (Flow/Search)}}}\overset{\text{Metric Collapse}}{\underset{\text{STDP}}{\to }}\underset{\beta_{0},\text{Low Entropy}}{\underset{︸}{\text{Assembly (Prior/Structure)}}}$$

In this view, a Prior is simply a fossilized Search path. The brain uses PNGs to explore the causal structure of the world (Time), and once validated, freezes them into the spatial architecture of columns (Space) to support rapid, zero-latency prediction.

## 5 Memory-amortized inference: topological predictive processing

We have established that the brain employs recursive condensation to manufacture topological separability. We now formally align this mechanism with the dominant theoretical framework of modern neuroscience: the Free Energy Principle (FEP) (Friston, 2010). We propose that minimizing variational free energy is mathematically equivalent to maximizing topological closure, and that this equivalence explains the scaling laws of cortical evolution.

### 5.1 Variational free energy as topological entropy

In the FEP framework, the brain minimizes an upper bound on surprise, known as Variational Free Energy (F): $F=\underset{\text{Divergence}}{\underset{︸}{D_{KL}\left(Q\left(s\right)||P\left(s|o\right)\right)}}-\underset{\text{Evidence}}{\underset{︸}{\ln\;P\left(o\right)}}$, where *Q*(*s*) is the recognition density (internal state) and *P*(*s*|*o*) is the posterior generative model. Classically, divergence represents a statistical mismatch. Topologically, we reinterpret high Free Energy as **Topological Entanglement**.

** Definition 2 (Topological Entanglement)**. Given a sensory input x∈X and two competing internal models (closed sets) *A* and *B*, the system is entangled if the input falls within the non-separable intersection of their open neighborhoods: *x*∈*U*_*A*_∩*U*_*B*_≠∅. In this state, no continuous separating function *f* exists, and the read-out is ambiguous (high entropy).

#### 5.1.1 The topological thesis of FEP

Minimizing Free Energy is the dynamical process of driving the system toward **Urysohn Disjointness**. When the brain updates its internal state (via gradient descent on F), it is performing a **Metric Condensation**: it contracts the open neighborhoods *U*_*A*_, *U*_*B*_ until the intersection vanishes (*U*_*A*_∩*U*_*B*_ = ∅). Therefore, surprise is simply the topological stress caused by leakage across decision boundaries. The brain minimizes surprise by condensing sensory data into disjoint, closed sets where linear separability is guaranteed.

#### 5.1.2 A topological field theory extension of the free energy principle

This geometric perspective naturally extends the Free Energy Principle (FEP) (Friston, 2010) into the domain of *topological field theory*. We propose that the brain minimizes an extended action functional comprising both the standard variational free energy (prediction error) and a penalty for *topological complexity* (e.g., the Euler characteristic χ Hatcher, 2005). Under this regime, the learning process described by TTT is formally equivalent to a symmetry-breaking event where the system tunnels from a high-energy metastable state, characterized by complex, disconnected topology (“holes” in understanding), to a low-energy vacuum state of trivial topology (the amortized scaffold). Consequently, the biological imperative is to act as a “Maxwell's Demon” of geometry (Maruyama et al., 2009): the system dissipates metabolic heat not merely to predict sensory data, but to actively reduce the topological entropy of the manifold, permanently converting the high cost of active search into the zero-cost inertia of structure.

### 5.2 The amortization cost theorem

#### 5.2.1 Evolution's cheat code: the predictive savitch machine

We can now synthesize the evolutionary logic of the mammalian neocortex. Biological survival requires predicting complex, chaotic futures (The Time Dragon). A naive solution, increasing the speed of neural firing, is metabolically impossible. The only viable alternative is to exploit the computational equivalence NPSPACE⊆PSPACE. Evolution selected for the neocortical sheet because it is a **Predictive Savitch Machine**.

1. **Input:** it takes problems that are exponentially hard in Time (Search).
2. **Process:** it applies Recursive Topological Condensation, freezing successful search paths into spatial hardware (Prior).
3. **Output:** it solves future instances of the problem in Polynomial Space (Navigation).

#### 5.2.2 Recursive condensation and Mountcastle's principle

Our framework reinterprets Mountcastle's columnar organization (Mountcastle, 1957) not merely as modular processing, but as the physical instantiation of *recursive topological quotienting*. We posit that every cortical column functions as a discrete Urysohn Operator: it ingests a high-dimensional, entangled input (Search/Open Set) at Layer IV, enforces separation via lateral inhibition (Urysohn Cut) at Layers II/III, and emits a condensed, zero-margin token (Navigation/Closed Set) at Layers V/VI. This explains the striking uniformity of cortical architecture: since the topological mechanism for separability (Urysohn's Lemma) is dimension-agnostic, the biological hardware implementing it must be invariant across scales. In the context of predictive processing (Keller and Mrsic-Flogel, 2018), this hierarchy acts as a *metric sieve*: each level of recursion filters out metric deformations (noise) via condensation, passing only topologically stable invariants to the level above. Therefore, the cortex breaks the Curse of Dimensionality by physically manufacturing a nested sequence of quotient spaces, $M_{\text{sensory}}\to \cdots \to M_{\text{concept}}$, where linear separability is maintained at every step.

If the brain minimizes free energy via condensation, how does this scale? We define **Memory-Amortized Inference (MAI)** as the strategy of storing these condensed states (priors) to avoid re-computing the search path (Gershman and Goodman, 2014). Consider a cognitive task requiring a search depth *L* (e.g., planning *L* steps ahead) in a state space with branching factor *b*. (1) **Cost of Search** (*C*_*search*_**):** without priors, the system must explore the tree. The complexity is exponential in time: *O*(*b*^*L*^), scaling as NPSPACE. (2) **Cost of Structure** (*C*_*space*_**):** the system stores intermediate condensed tokens (cortical columns). The complexity is linear in space: *O*(*N*), where *N* is the number of columns.

** Theorem 2 (Amortization Cost Theorem)**. Let R be the *Predictive Reach* of the system (the maximum depth *L* traversable without divergence). Under MAI, a linear increase in structural capacity (space *N*) yields an exponential increase in predictive reach. $R\left(N\right)\propto b^{\alpha N}$ where α represents the efficiency of the Urysohn quotient map (the compression ratio of the column).

#### 5.2.3 Expansion: the spatiotemporal exchange rate

Theorem 2 provides the rigorous justification for the “linear hardware, exponential software” observation: the brain trades *space* (adding columns) to purchase *time* (computational depth). Formally, the theorem establishes that the storage complexity of the manifold |*N*(ϵ)| scales exponentially with the intrinsic dimension *d*. In a fixed-resource system, reducing the resolution error ϵ typically requires a corresponding increase in temporal processing (iterations of the search loop) to resolve ambiguity. However, by expanding the physical substrate, linearly adding cortical columns to increase the covering number |*N*(ϵ)|, the system can physically instantiate a finer ϵ-net. This spatial expansion effectively pre-computes the higher resolution, converting what would be a costly, deep-temporal search (high computational depth) into a shallow, constant-time routing operation across a larger static scaffold. In summary, the biological strategy of massive parallelism is not merely about throughput, but about *thermodynamic amortization*: investing linear metabolic maintenance costs (Space) to bypass the exponential energy costs of dynamic error correction (Time).

## 6 Discussion

We have proposed that the mammalian neocortex operates as a *biological Savitch machine*, employing *recursive condensation* to solve the dual challenges of combinatorial complexity (The Time Dragon) and dimensional sparsity (The Space Dragon). This framework moves beyond metaphor, offering specific, falsifiable predictions about neural dynamics and providing a unified ontology for intelligence.

### 6.1 The unification

This paper began with a question: How does a finite biological engine achieve exponential cognitive reach? The answer lies in the isomorphism between distinct fields of mathematics, computation, and neurobiology. Our framework unifies them into a single coherent narrative of intelligence (Table 2).

**Table 2:** The grand unification of recursive condensation.

**Discipline**	**Core principle**	**Role in cognition**
Topology	Urysohn's Lemma	**The condition:** separability requires disjoint closure.
Complexity	Savitch's Theorem	**The algorithm:** time (Search) can be traded for Space (Recursion).
Neuroscience	Mountcastle/PP	**The hardware:** columns instantiate the recursive collapse.
Thermodynamics	Dark energy & Spontaneous Slow Oscillations (SSOs)	**The engine:** energy maintains topology; SSOs clock the recursion.

The convergence of three clues in topology, complexity, and neuroscience offers new insight to the paradox of intelligence.

**Topology provides the Why**. Urysohn's Lemma explains why the brain discards information. It is not compression for bandwidth's sake but *condensation for separability's sake*. The brain must collapse the metric manifold to separate and stabilize topological concepts.

**Complexity provides the How**. Savitch's Theorem explains why the brain is hierarchical. A deep stack of cortical columns is not just extracting features; it is a *recursion stack* that allows the brain to emulate nondeterministic search (NPSPACE) using deterministic, spatially constrained hardware (PSPACE).

**Neuroscience provides the What**. Predictive Processing explains the structural dynamic. The brain is not a passive filter but an active *metric sculptor*. By minimizing Free Energy, it is physically maximizing the topological disjointness of its internal states, continuously shaping the manifold to reduce future surprise.

**Thermodynamics provides the engine and the clock**. The computational logic of this framework requires a physical mechanism to drive its iterations, which is resolved by the brain's Spontaneous Slow Oscillations (SSOs) (Batterink et al., 2016; Timofeev et al., 2000) and its massive baseline metabolic cost, often termed the brain's dark energy (Gong and Zuo, 2025; Raichle et al., 2001). Under our framework, the SSO cycle acts as the physical clock for the *Parity Alternation Principle*. The cortical “Down-state” (hyperpolarization) corresponds strictly to the *Quotienting (Reset)* operation (Dang-Vu et al., 2008)—wiping away the transient metric noise to return the system to the null manifold. The subsequent “Up-state” (depolarization) corresponds to *fiber activation*, engaging the topological scaffold to integrate new sensory coordinates (Robinson and Roy, 2015). Importantly, the massive dark energy expenditure is the thermodynamic cost of fighting the Second Law of Thermodynamics to maintain this *topological scaffold*. Without this constant injection of energy, the stable semantic graph (the topology) would spontaneously decay into disorganized episodic noise (metric entropy) (Robinson et al., 2015). Therefore, recursive condensation is not merely a mathematical algorithm, but a thermodynamically mandated process of structural maintenance (Pang et al., 2023).

### 6.2 Predictions: the biological and computational implementation

If the cortex functions as a recursive Urysohn operator, specific physiological rhythms must correspond to the topological phases of expansion (search) and contraction (closure).

#### 6.2.1 Prediction 1: gamma oscillations as the Urysohn clock

We posit that the **Pyramidal-Interneuron Gamma (PING)** rhythm (Traub et al., 1997) is the physical manifestation of the Parity Alternation Principle. *Excitation phase (odd/*β_1_*):* pyramidal firing corresponds to the definition of the open neighborhood *U* (metric expansion/search). *Inhibition phase (even/*β_0_*):* The subsequent volley of Parvalbumin+ interneurons corresponds to the Urysohn Cut, enforcing the closure $\bar{U}$ (metric condensation). *Testable implication:* pathologies involving Gamma dysregulation (e.g., Schizophrenia Insel, 2010) should be reinterpreted not as signaling failures, but as *Topological Leakage*. A failure of the inhibitory cut results in *U*∩*B*≠∅, leading to the entanglement of distinct concepts (hallucinations) and the inability to distinguish internal priors from external sensory flow (Fletcher and Frith, 2009).

**Integration with macroscopic predictive coding:** while the Gamma PING rhythm executes the local topological cut (Urysohn Condensation), we map this to the global predictive coding dynamics mediated by Alpha-band traveling waves (Alamia and VanRullen, 2019). In our framework, top-down Alpha waves deliver the topological prior (the intended boundary of *U*), while the local Gamma circuit executes the metric cut. Cross-frequency phase coupling ensures the local Parity Alternation is synchronized with the global predictive cascade. Consequently, the topological leakage seen in Schizophrenia (Insel, 2010) is the mechanical failure of the Gamma Urysohn cut to separate top-down Alpha predictions from bottom-up sensory metrics, causing internal priors to be erroneously broadcast as external sensory realities (Sterzer et al., 2018).

#### 6.2.2 Prediction 2: sleep and SWRs as offline closure

While Gamma manages micro-scale condensation (Buzsáki and Wang, 2012), global topological consolidation requires offline processing to prevent interference between the open sensory stream and the closed structural priors. We predict that *Sharp-Wave Ripples (SWRs)* during Slow-Wave Sleep represent the system performing *Global Closure* (Buzsáki, 2015). During waking, the brain accumulates high-entropy search paths (PNGs/β_1_ traces). During sleep, these temporal loops are replayed and collapsed via STDP into permanent structural weights (Columns/β_0_). *Testable Implication:* deprivation of SWRs should specifically impair *transitive inference* (the ability to link *A*→*B*→*C* via a structural backbone), as the system fails to memoize the search paths into navigable geometry (Buzsáki and Moser, 2013).

**Integration with the SSO Spectral Hierarchy**: while Gamma provides the Urysohn Clock for local, columnar condensation, global structural updates require a macroscopic parity operator. We propose that the fractal nature of brain oscillations reflects a hierarchy of topological closure (Staresina et al., 2015). Hippocampal SWRs, the fast, metric replays of waking search paths, do not permanently alter the cortical manifold on their own. Instead, they must be temporally nested within the Up-states of standard Slow Oscillations (~1 Hz), which are themselves amplitude-modulated by infra-slow rhythms such as the *Slow-4 band (0.01–0.027 Hz)* (Lecci et al., 2017). In this framework, Slow-4 acts as the transitional band for global closure. It provides the prolonged temporal window of brain-wide coherence necessary to quotient disparate local replays into a unified semantic manifold. Consequently, disruptions to the hierarchical nesting of SWRs within the Slow-4 envelope will specifically fracture *transitive inference* (*A*→*B*→*C*), as the brain loses the macroscopic parity state required to stitch local metric sequences into a globally navigable topology (Robinson et al., 2015).

#### 6.2.3 Prediction 3: SNNs as pulse-position Urysohn operators

We predict that the recursive Urysohn operator is implemented via Time-to-First-Spike (TTFS) coding within a delay-coupled Spiking Neural Network (SNN) (Ghosh-Dastidar and Adeli, 2009). In this regime, the metric distance between two topological sets is encoded as the relative phase lag between spike volleys. (1) *Latent Warp via Synaptic Delays:* The metric slingshot is physically realized by the modulation of dendritic delays (Muller et al., 1996). By shortening the delay Δ*t* along the principal axis of fluidity (GHL discovery), the SNN collapses the temporal distance between the agent's state and the goal; (2) *The Operator Mechanism:* The Urysohn Cut is implemented by a coincidence detection threshold. Only spikes arriving within a narrow ϵ-window trigger the downstream token (a columnar burst), effectively performing a non-linear contraction of high-dimensional input into a single point in the next layer. *Testable Implication:* We predict that in navigating topological traps, successful learning will be marked by a temporal compression of the spike train. As the Maze becomes a Bowl, the sequence of spikes representing the path will move from a sparse, high-entropy temporal distribution to a synchronous, low-latency volley. Computational models of SNNs using surrogate gradient descent (Neftci et al., 2019) should show that incorporating a metric slingshot penalty (rewarding temporal sparsity) significantly reduces the time-to-solution in non-convex landscapes.

## 7 Conclusion

The intelligence of the mammalian brain does not arise from raw speed (defeating the Time Dragon directly) nor from infinite storage (defeating the Space Dragon directly). It arises from the geometry of the compromise. By implementing MAI, the cortex cheats the laws of complexity. It builds a *biological Savitch machine* that converts the impossible exponential explosion of the future into the manageable polynomial architecture of space. By enforcing Urysohn's condition of disjoint closure, the brain transforms exponential, nondeterministic search into deterministic, serial traversal, an embodied realization of Savitch's theorem. Using the hippocampus for topological indexing, the cortex slays the *Space Dragon* (dimensionality) through structural reuse and taming the *Time Dragon* (combinatorial explosion) through serial amortization. Under our framework, predictive processing is reinterpreted as *recursive condensation*. Each cortical column acts as a topological operator, collapsing high-entropy temporal flows into stable spatial invariants. This tradeoff, linear cortical expansion yielding exponential representational reach, explains the evolutionary advantage of the mammalian neocortex. In conclusion, the mammalian brain is a machine that cheats the limits of metabolic energy by exploiting the geometry of the quotient map.

More broadly, MAI reframes intelligence as a principle of *topological inference*: cognition is not fast parallel search, but the recursive enforcement of closure until context and content cohere into a self-consistent, persistent structure. This bears critical implications for the future of Artificial General Intelligence (AGI). Statistical oracle-based approaches, such as Large Language Models (LLMs), attempt to solve the Space Dragon by simply inflating it, scaling parameters to memorize the manifold, while ignoring the Time Dragon. They seek to be instantaneous oracles. However, our analysis suggests that such approaches are theoretically bounded. A true AGI will not be an instantaneous oracle, but a *serial thinker*. It must respect the fundamental constraint that **P**≠**NP**. Like the human mind, it must take the *time* to think, engaging in the recursive, temporal condensation of information to navigate the exponential complexity of the real world.

## Data Availability

The original contributions presented in the study are included in the article/supplementary material, further inquiries can be directed to the corresponding author.
